# The Coordination of Gene Expression within Photosynthesis Pathway for Acclimation of C_4_ Energy Crop *Miscanthus lutarioriparius*

**DOI:** 10.3389/fpls.2016.00109

**Published:** 2016-02-09

**Authors:** Shilai Xing, Lifang Kang, Qin Xu, Yangyang Fan, Wei Liu, Caiyun Zhu, Zhihong Song, Qian Wang, Juan Yan, Jianqiang Li, Tao Sang

**Affiliations:** ^1^State Key Laboratory of Systematic and Evolutionary Botany, Institute of Botany, Chinese Academy of SciencesBeijing, China; ^2^University of Chinese Academy of SciencesBeijing, China; ^3^Key Laboratory of Plant Resources and Beijing Botanical Garden, Institute of Botany, Chinese Academy of SciencesBeijing, China; ^4^Key Laboratory of Plant Germplasm Enhancement and Specialty Agriculture, Wuhan Botanical Garden, Chinese Academy of SciencesWuhan, China

**Keywords:** C_4_ photosynthesis, acclimation, expression coordination, cyclic electron transport, water use efficiency

## Abstract

As a promising candidate for the second-generation C_4_ energy crop, *Miscanthus lutarioriparius* has well acclimated to the water-limited and high-light Loess Plateau in China by improving photosynthesis rate and water use efficiency (WUE) compared to its native habitat along Yangtze River. Photosynthetic genes were demonstrated as one major category of the candidate genes underlying the physiological superiority. To further study how photosynthetic genes interact to improve the acclimation potential of *M. lutarioriparius*, population expression patterns within photosynthesis pathway were explored between one mild environment and one harsh environment. We found that 108 transcripts in assembled transcriptome of *M. lutarioriparius* were highly similar to genes in three Kyoto Encyclopedia of Genes and Genomes (KEGG) photosynthesis pathways of sorghum and maize. Phylogenetic analyses using sorghum, maize, rice, and *Arabidopsis* genes of dark reaction identified 23 orthologs and 30 paralogs of *M. lutarioriparius* photosynthetic genes. These genes were also clustered into two kinds of expression pattern. 87% of transcripts in dark reaction were up-regulated and all 14 chloroplast-encoded transcripts in light reaction increased degradation in the harsh environment compared to the mild environment. Moreover, 80.8% of photosynthetic transcripts were coordinated at transcription level under the two environments. Interestingly, LHCI and PSI were significantly correlated with F-ATPase and C_4_ cycle. Overall, this study indicates the coordinated expression between cyclic electron transport (consisting of LHCI, PSI, and ATPase) and CO_2_-concentrating mechanism (C_4_ cycle) could account for photosynthesis plasticity on *M. lutarioriparius* acclimation potential.

## Introduction

It has been attracting increasing interest in recent years for studying the high crop productivity potential of C_4_ pathway of photosynthesis in the face of population pressure along with global warming (Dohleman and Long, 2009; Zhu et al., 2010; Butler and Huybers, 2012). Many C_4_ plants of the NADP-malic enzyme (NADP-ME) subtype have been domesticated and represent irreplaceable sources of food, biomass, and bioenergy. For instance, *Miscanthus* is the promising bioenergy crop in the marginal land and has huge biomass due to the high level of photosynthetic rate (Sang, 2011; Sang and Zhu, 2011; Liu et al., 2012, 2014; Liu and Sang, 2013; Mi et al., 2014). While the adaptation of NADP-ME C_4_ plants to high temperatures and high light intensities is well established and understood (Hattersley, 1983; Hatch, 1987; von Caemmerer and Furbank, 2003; Sage, 2004; Edwards et al., 2010), it is not clear how, and to what extent, C_4_ plant acclimates to changes in growing environments.

Many studies have been conducted to understand the expression pattern of photosynthesis genes or proteins in maintaining high photosynthetic rates during the cold acclimation of a triploid hybrid, *Miscanthus* × *giganteus*, which originated from a cross between diploid *Miscanthus sinensis* and tetraploid *Miscanthus sacchariflorus* (Naidu and Long, 2004; Farage et al., 2006; Friesen et al., 2014; Glowacka et al., 2014; Peixoto et al., 2015). Naidu et al. (2003) found that protein amounts and transcript abundance of pyruvate phosphate dikinase (PPDK) and Rubisco large subunit (rbcL) were stabilized both at 25/20 and 14/11°C (day/night), while those of phosphoenolpyruvate carboxylase (PEPC) varied little due to growth temperature. Thus, they hypothesized that the maintenance of PPDK and rbcL amounts in *M.* × *giganteus* are especially important in maintaining high rates of C_4_ photosynthesis at low temperature. However, the catalytic properties of purified Rubisco and Rubisco in crude leaf extracts differ non-significantly between *M.* × *giganteus* and maize grown at 14°C, which temperature could lead to high photosynthetic rate for *M.* × *giganteus* in relative to maize. Therefore, low temperature acclimation of *M.* × *giganteus* with high photosynthetic rate does not result from the catalytic properties of Rubisco (Wang et al., 2008a). During the temperature transition from growing at 25–14°C, protein content of PPDK transiently declines but then steadily increases, meanwhile, the extractable PPDK activity in warm-grown leaves is lower than in cold-grown leaves (Wang et al., 2008b). It indicates the cold acclimation role of PPDK in dark reaction. Besides, the transcriptional abundance of chlorophyll a/b-binding protein CP29 (lhcb4), Chlorophyll a-b binding protein CP26 (lhcb5), NADH dehydrogenase F (ndhF), ATP synthase alpha subunit (atpA), Photosystem II protein D1 (psbA), and Cytochrome f (petA) in light reaction are increased in *M.* × *giganteus* after 14 days of chilling (14°C). Consistently, the psbA protein and LHCII type II chlorophyll a/b-binding protein also show significant increases in *M.* × *giganteus* during chilling. Thus, *M.* × *giganteus* seems to increase mRNA levels of photosynthetic proteins and proteins protecting photosystem II for their synthesis to counteract their loss in chilling conditions (Spence et al., 2014).

In studying the acclimation of *M.* × *giganteus* to transient changes in light quality, transient changes in light treatments quickly lower the rate of net CO_2_ assimilation and disrupt the coordination of C_3_ and C_4_ cycles, which potentially seems to affect cyclic electron flux around photosystem I and chloroplast rearrangement (Sun et al., 2012, 2014). Meanwhile, *M.* × *giganteus* acclimates poorly to low water availability (Clifton-Brown and Lewandowski, 2000) and photosynthetic performance is reduced by a decrease in stomatal conductance, which is followed with a decrease in chlorophyll content and chlorophyll fluorescence (Ings et al., 2013).

In facing the combination of various novel ecological factors in the Loess Plateau, *Miscanthus lutarioriparius* shows excellent performance like *M.* × *giganteus* in acclimating to low temperature, but unlike *M.* × *giganteus* it appears to well tolerate water deficit and high light with improved WUE and photosynthesis rate. *Miscanthus lutarioriparius* has higher survival rate and produced higher biomass yield than *M. sinensis* and *M. sacchariflorus* when planted in the Loess Plateau of China (Yan et al., 2012). It is endemic in central China (Yan et al., 2015a), like Jiangxia of Hubei Province (JH), where the average of the monthly average precipitation, the monthly average temperature and the monthly total hours of sunshine are 140.8 mm, 20.2°C, and 147.5 h, respectively. When transplanted to Qingyang of Gansu Province (QG) located in the Loess Plateau, where the average of the monthly average precipitation, the monthly average temperature and the monthly total hours of sunshine are 55.0 mm, 12.3°C, and 194.4 h during its entire growing season, the stomatal conductance (*g*_*s*_) of the species is significantly lower throughout the entire growing season, while the photosynthetic rate and the instantaneous water use efficiency (WUE) are higher, and almost triple at the late growing stage (Yan et al., 2015b). Eight photosynthesis genes are identified to be related to WUE through the transcriptome-wide matrix correlation analysis, and the ability to maintain or improve the photosynthesis rate under the condition of reduced stomatal transpiration may explain the consistently higher physiological WUE for *M. lutarioriparius* in water-limited environments (Fan et al., 2015). However, the patterns of gene interaction existing in the photosynthesis pathway of *M. lutarioriparius* remain largely unknown. Especially, it is valuable to elucidate how the patterns influence acclimation potential of this energy crop in the newly dry, cold and overly sunlit environment.

The RNA-seq method provided a direct and efficient way to explore the complex mechanism of C_4_ biochemistry and tissue structure underlying eco-physiological change in *Miscanthus* species without any reference genome (Chouvarine et al., 2012; Barling et al., 2013; Kim et al., 2014). RNA-seq technique is used to compare the whole genome variation of expression for the random sampled individuals from two contrasting sites, JH and QG (Xu et al., 2015). 78 transcripts associated with the subunits of photosystem and the enzymes of carbon fixation are identified from the *de novo* assembled transcriptome of *M. lutarioriparius* population using homologous search and phylogenetic methods. Then through analyzing the correlated expression pattern of 78 transcripts from two contrasting experimental fields, the results show that at the transcription level of photosynthesis pathway the coordinated expression of transcripts involved in light-harvesting chlorophyll-a/b protein of photosystem I (LHCI), photosystem I (PSI), F-ATPase, Calvin and C_4_ cycles could synchronize the first step of activities of cyclic electron transport and CO_2_-concentrating mechanism of NADP-ME type. The down-regulation of transcripts involved in cyclic electron transport and the up-regulation of transcripts in CO_2_-concentrating mechanism characterize *M. lutarioriparius* with improved WUE in the cold, dry, and overly sunlit environment.

## Materials and methods

### Sampling and transcriptome assembling

Seeds were collected from the natural populations of *M. lutarioriparius*, and planted in two experimental fields, QG and JH in China, in 2009 (Yan et al., 2012). From the planted populations of *M. lutarioriparius*, 39 individuals were randomly sampled from each field site (Xu et al., 2015). The fourth leaf from the top of each individual was cut and immediately placed in liquid nitrogen. The samples were taken around noon on June 12th, 2012 in JH and on July 13th, 2012 in QG. On the basis of data gathered from the 2009-2011 growing seasons, the growing seasons of *M. lutarioriparius* (from seedling germination to flowering) were during the period of the later of March to October in JH and the later of April to November in QG, respectively (Yan et al., 2012). Thus, the growing season was about 1 month later in QG than in JH, which is consistent with the temperature patterns between the two locations. Therefore, by sampling with 1 month apart, it was the way to synchronize the developmental stages under natural conditions between the two field sites.

Total RNA of each leaf sample was isolated and purified using Trizol reagent (Invitrogen) and the RNeasy Mini kit (Qiagen), respectively (Xu et al., 2015). The oligo d (T) beads [Dynabeads® mRNA Purification Kit (Invitrogen)] was used to isolate mRNA using one round of purification (Xu et al., 2015). The 100 bp paired-end library was constructed for the isolated mRNA of each sample using NEBNext mRNA Library Prep Reagent Set for Illumina (NEB).

### Expression estimating and calculation of genetic diversity

The sequenced reads of each sample from the Illumina HiSeq 2000 were used to calculate the expression abundance (fragments per kilobase of transcript per million fragments mapped, abbreviated FPKM) of each *de novo* assembled transcript (TSA accession no. GEDE00000000; Xu et al., 2015). RNA-seq can capture mature nuclear mRNA and degraded chloroplast mRNA, both of which have 3′-end polyadenylation (Schuster et al., 1999). The steady-state level of chloroplast mRNA is not related to transcription rate but related to degradation rate (Rapp et al., 1992; Kim et al., 1993). Thus, FPKM is representative of the expression level of nuclear encoded mRNA and degradation level of chloroplast encoded mRNA.

Single nucleotide polymorphisms (SNPs) between samples were detected using SAMtools with default settings (Xu et al., 2015). Genetic diversity (π) was then calculated for each transcript in JH and QG based on SNPs using custom Perl script according to the method introduced by Nei and Li (1979) and (Xu et al., 2015).

### *In silico* identification of candidate transcripts involved in photosynthesis

The genes involved in *Zea mays* and *Sorghum bicolor* light and dark reactions were found in the Kyoto Encyclopedia of Genes and Genomes (KEGG) pathway (http://www.genome.jp/kegg/) including map00196, map00195 and map00710 (Table S1). The nucleotide sequences of these genes were downloaded from NCBI (http://www.ncbi.nlm.nih.gov/), and used to blast the assembled transcriptome sequences of *M. lutarioriparius* using BLASTN (Camacho et al., 2009) with e-value of e^−60^ and identity of 85%. The hit pairs between *M. lutarioriparius* and *Z. mays* or *S. bicolor* were summarized in Table S2. Through carefully scrutinizing the alignments of the hit pairs with the same target in Phytozome 9.1 (http://www.phytozome.net/), *MluLR17695* and *MluLR11855* were aligned to *XM_002437220.1 (Sobic.010G188300*), and merged as *MlFBA1*; *MluLR859*, and *MluLR11354* were aligned to *XM_002437717.1 (Sobic.010G023700)*, and merged as *MlTKL1*; *MluLR17298*, and *MluLR15467* were aligned to *NM_001112268.1* (*GRMZM2G306345*), and merged as *MlPPDK1* (Figure S1). The coverage of each candidate transcript was calculated as the ratio of alignment length to the length of *M. lutarioriparius* sequence. The similarity was calculated as the average identity of all hits within one sequence pair.

Transcripts of C_4_ enzymes used by Wang et al. (2009) and Calvin enzymes used by Reyes-Prieto and Troncoso-Ponce in dark reaction as well as their isoforms in *S. bicolor, Oryza sativa, Z. mays*, and *Arabidopsis thaliana* (Reyes-Prieto and Bhattacharya, 2007; Troncoso-Ponce et al., 2012) were downloaded from NCBI or Phytozome 9.1. Besides, Transcripts of three species, *O. sativa, Z. mays*, and *A. thaliana*, in relative to Calvin enzyme families collected in PMN (http://www.plantcyc.org/) were downloaded from Phytozome 9.1. Transcripts of five species were aligned separately in each family of eight Calvin cycle families, D-ribulose-5-phosphate 3-epimerase (RPE), ribose-5-phosphate isomerase (RPI), phosphoglycerate kinase (PGK), glyceraldehyde-3-phosphate dehydrogenase (GAPDH), phosphoribulokinase (PRK), transketolase (TKL), fructose- bisphosphate aldolase (FBA), and triose phosphate isomerase (TPI), and four C_4_ families, carbonic anhydrase (CA), NADP-malate dehydrogenase (NADP-MDH), PEPC, and PPDK. Transcripts of two families sharing the common ancestor, fructose bisphosphatase (FBP) and sedoheptulose-bisphosphatase (SBP), were aligned together. The highly divergent transcripts of family NADP-malic enzyme (NADP-ME) were aligned in ClustalX 1.83 (Chenna et al., 2003), and sent to Gblocks Server (http://molevol.cmima.csic.es/castresana/Gblocks_server.html) for selecting the conserved blocks with the more stringent criteria, i.e., at least eight sequences for a conserved position, at least 12 sequences for a flanking position, at most four contiguous non-conserved position, at least 10 bps for a block, and none allowed gap positions.

All above alignments were used to construct phylogenetic trees using the neighbor-joining methods in MEGA 5.0 (Tamura et al., 2011). We sought to differentiate C_4_ enzymes and their C_3_ progenitors in *M. lutarioriparius*, by comparing key C_4_ photosynthetic enzyme genes of *M. lutarioriparius* with those of other C_4_ and C_3_ plants. We used the 20 transcripts of five families from *M. lutarioriparius* to elucidate the phylogeny of C_4_ cycle enzymes with molecular data from *A. thaliana, O. sativa, Z. mays*, and *S. bicolor*. The annotation of cellular location and molecular function for enzymes of *A. thaliana* and *O. sativa* was obtained by querying TAIR (http://www.arabidopsis.org/) and RGAP (http://rice.plantbiology.msu.edu/index.shtml), respectively. The plastid-labeled *A. thaliana* genes were further searched in AraCyc Pathways database (http://www.arabidopsis.org/biocyc/index.jsp). To gain a clearer picture of Calvin cycle enzymes of *M. lutarioriparius*, it is critical to analyze the genome data from the higher plants that presumably share the same origin and diversification of plastidic enzymes and cytosolic enzymes. We used the 32 transcripts of ten families, with the exception of the single transcript of rbcL from *M. lutarioriparius*, to elucidate the phylogeny of Calvin cycle enzymes with molecular data from *A. thaliana, O. sativa, Z. mays*, and *S. bicolor*.

### Statistic test of expression difference between different gene sets

One nonparametric test, the two-sample Kolmogorov–Smirnov test, is used to test whether two distributions are the same. Two gene sets, 23 transcripts of dark reaction and 32 transcripts of non-dark reaction (paralogs of genes in dark reaction), were tested for the expression median of each transcript in JH and QG, respectively. Furthermore, the same two gene sets were tested for the ratio of the expression median of each transcript between QG and JH. Another non-parametric test, the Wilcoxon rank sum test, is used to assess whether two population mean ranks differ. It was carried out for two gene sets, five transcripts of the C_4_-specific cycle and 18 transcripts of the Calvin cycle using the expression median of each transcript in QG. It was also carried out for two gene sets of five transcripts in ATP-related group and 15 transcripts in Other group, as well as three transcripts in NADPH-related group and 15 transcripts in Other group, using the ratio of the expression median of each transcript between QG and JH.

### Clustering and correlation analysis of gene expression of photosynthesis pathway

For three combined transcripts, their values of gene expression were recalculated as the average level of two parts in each sample. Fifty-five transcripts functioned in light reaction and fifty-three transcripts in dark and non-dark reactions were by themselves clustered by both genes and samples. The clustering procedure, based on the centroid linkage method using population FPKMs (Table S3) after log_2_ transformation, centering genes at median, normalizing genes, centering arrays at median, and normalizing arrays in Cluster 3.0 (Eisen et al., 1998). The cluster results were displayed in Treeview v1.60 (Page, 1996).

The Spearman's correlation was conducted for every two transcripts of 78 transcripts using 78 FPKMs of both sites. The seven functional groups in light reaction were defined according to KEGG pathway map00196 and map00195, i.e., LHCII consisting of Lhcbs, PSII consisting of Psbs, LHCI consisting of Lhcas, PSI consisting of Psas, cyt *b*_6_*/f*, PET, and F-ATPase, and the two functional groups in dark reaction were defined as C_4_ cycle and Calvin cycle. To measure the relatedness within each functional group or between two functional groups, the absolute values of Spearman's rank correlation coefficients (rho) of related transcript pairs were averaged. To test whether the relatedness within each group or between groups, was significantly larger than the baseline of the light and dark reactions, absolute values of rho of related transcript pairs were compared with 0.23 (the median of the absolute values of all coefficients as the background) using the Binomial Test.

## Results

### Candidate photosynthesis transcripts of *M. lutarioriparius* population

**Figure 1:**
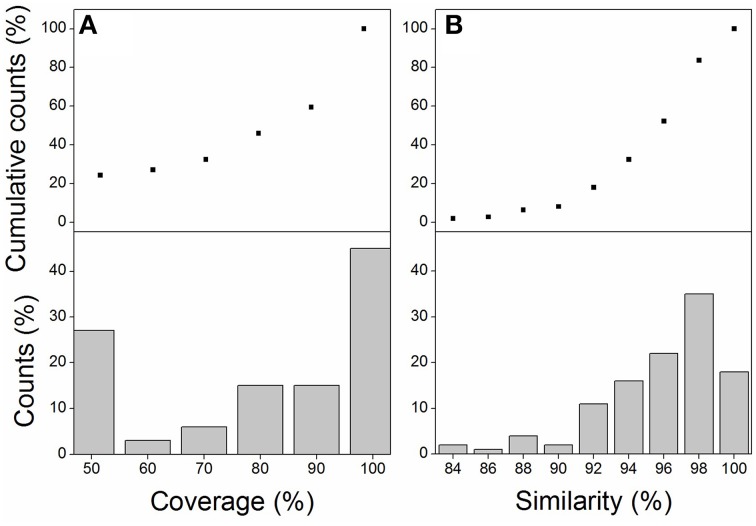
**The coverage distribution (A) and similarity distribution (B) of candidate photosynthesis transcripts from *M. lutarioriparius* transcriptome relative to the best hit transcripts of *Z. mays* or *S. bicolor* genome**.

One-Hundred and eight transcripts were identified by local blasting the *M. lutarioriparius* assembled reference sequences (Xu et al., 2015) with *Z. mays* genes and *S. bicolor* genes in relative to the components of KEGG photosynthesis pathway (Table S1). The best hits between *M. lutarioriparius* and *Z. mays* or *S. bicolor* were listed in Table S2. Sixty-eight percent of transcripts with >70% coverage and 90% transcripts with >90% similarity were observed in candidate photosynthesis transcripts of *M. lutarioriparius* relative to *Z. mays* or *S. bicolor* sequences (Figures 1A,B). These transcripts include 55 members referred as 41 components in light reaction, and 53 members referred to 16 enzymes in dark reaction (Tables S1, S2).

### The phylogenetic identification of transcripts in calvin cycle and C_4_ cycle

**Figure 2:**
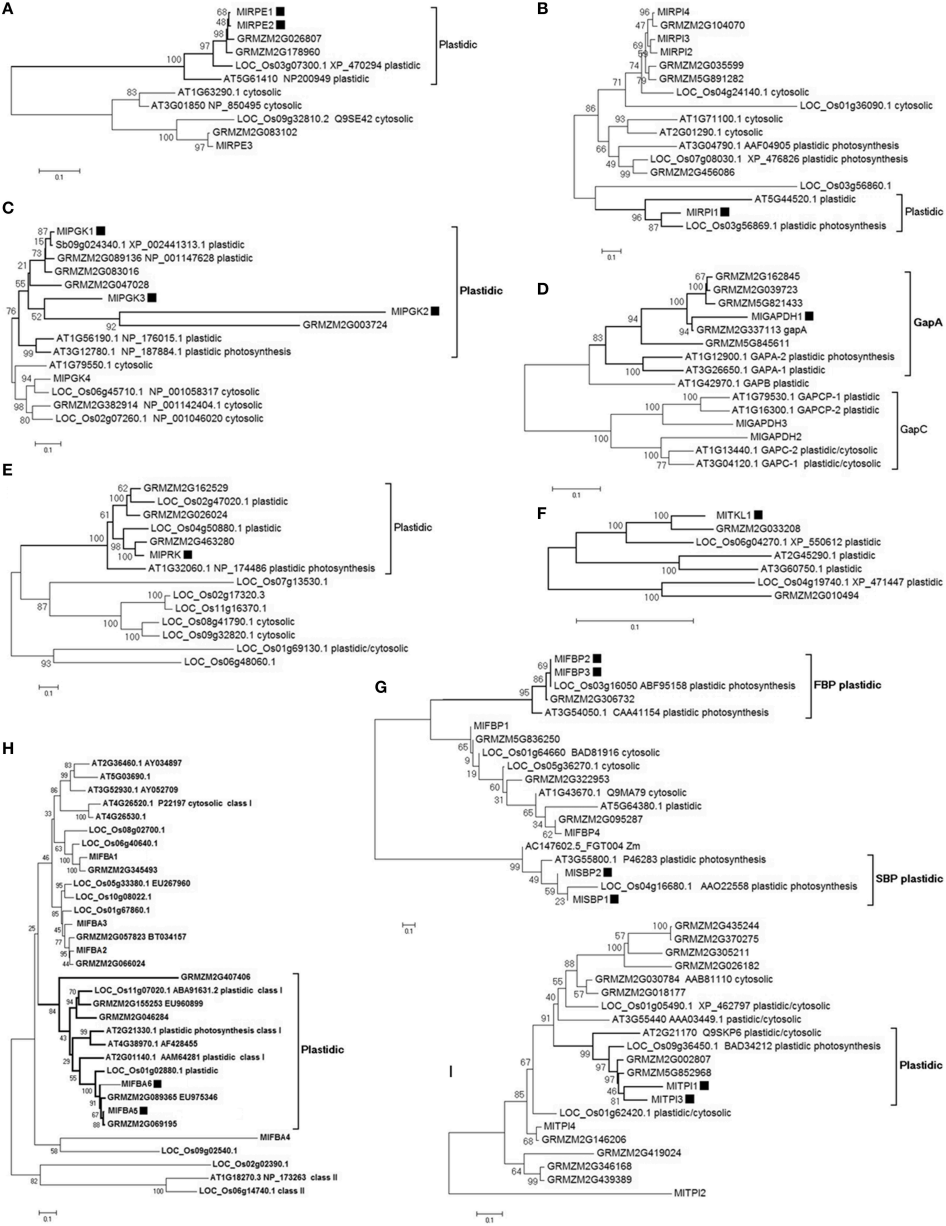
**Neighbor-joining trees of Calvin enzyme transcripts and their isoforms in *M. lutarioriparius*, *S. bicolor*, *Z. mays*, *O. sativa*, and *A. thaliana***. Bootstrap percentage values in 500 times are shown as integers. Black square shows the identified *M. lutarioriparius* transcript involved in Calvin cycle. In the transcript IDs, Ml indicates *M. lutarioriparius*, Sb indicates *S. bicolor*, Os indicates *O. sativa*, GRMZM indicates *Z. mays*, and AT indicates *A. thaliana*. **(A)** RPE, D-ribulose-5-phosphate 3-epimerase; **(B)** RPI, ribose-5-phosphate isomerase; **(C)** PGK, phosphoglycerate kinase; **(D)** GAPDH, glyceraldehyde-3- phosphate dehydrogenase; **(E)** PRK, phosphoribulokinase; **(F)** TKL, transketolase; **(G)** FBP and SBP, fructose bisphosphatase and Sedoheptulose-bisphosphatase; **(H)** FBA, fructose- bisphosphate aldolase; **(I)** TPI, triose phosphate isomerase.

The eukaryotic Calvin cycle involves 11 different enzymes that are nuclear encoded and plastid targeted to express their function, with the exception of rbcL that is encoded in the plastid genome in higher plants. Photosynthetic eukaryotes also contain cytosolic enzymes involved in glycolysis and gluceoneogenesis that are homologous to enzymes of Calvin cycle. They catalyze reactions similar to those in the Calvin cycle, and were present in eukaryotes before plastid origin (Martin and Schnarrenberger, 1997). The Neighbor-joining phylogenetic analyses using full-length transcripts showed that all ten families have distinguished the plastid target enzymes from the cytosolic enzymes (Figure 2). Except for *AT1G79530.1, AT1G16300.1, AT1G13440.1*, and *AT3G04120.1* in the GapC branch (Figure 2D), the plastid-labeled *A. thaliana* genes were annotated to be involved in Calvin cycle in AraCyc Pathways database. In the trees of eight families, the plastid labeled branches with high support values have *Arabidopsis* enzymes or/and *Oryza* enzymes annotated as functioning in Calvin cycle (Figures 2B–E,G–I), which indicated other enzymes in those branches would also function in Calvin cycle. Based on the plastid target character (nuclear encoded and plastid targeted to express their function) and the known function of orthologs in *Arabidopsis* or/and *Oryza*, 17 *M. lutarioriparius* transcripts were identified to be possible Calvin cycle enzymes (black squares in Figure 2). One *MlrbcL* transcript has 484 aa, and has four best-hits with similar length of 483 aa and similar identity of 99.2% in *Z. mays* (GRMZM2G360821_T01, GRMZM2G385635_T01, GRMZM2G448344_T01, GRMZM5G815453_T01), which are ribulose bisphosphate carboxylase large chain proteins.

**Figure 3:**
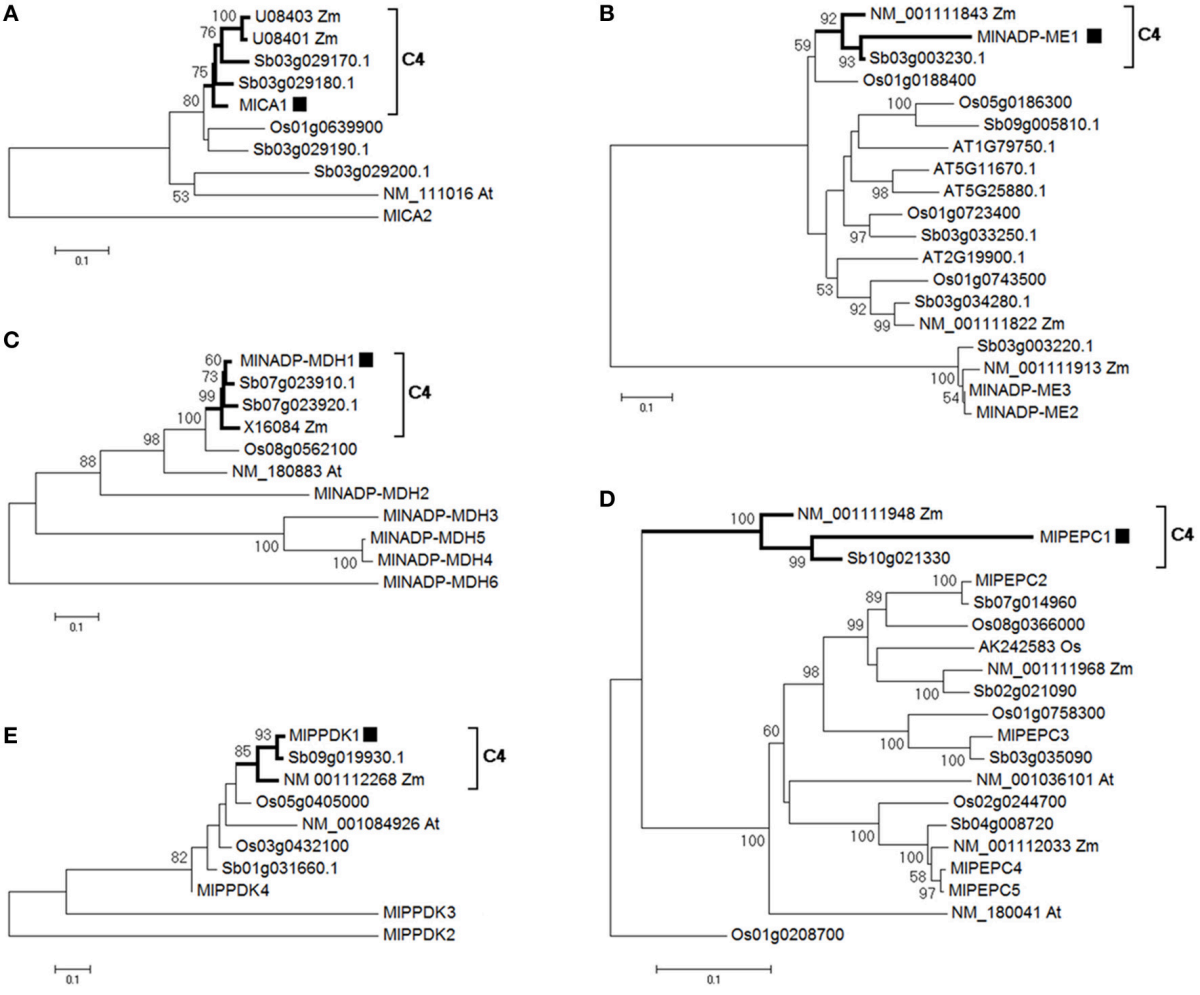
**Neighbor-joining trees of C_4_ enzyme transcripts and their C_3_ isoforms in *M. lutarioriparius*, *S. bicolor*, *Z. mays*, *O. sativa*, and *A. thaliana***. Thick branches and brackets show the C_4_ transcripts. Bootstrap percentage values in 500 times are shown as integers. Black square shows the identified *M. lutarioriparius* transcript involved in C_4_ cycle. In the transcript IDs, Ml indicates *M. lutarioriparius*, Sb indicates *S. bicolor*, Os indicates *O. sativa*, Zm indicates *Z. mays*, and At indicates *A. thaliana*. **(A)** CA, carbonic anhydrase; **(B)** NADP-ME, NADP-malic enzyme; **(C)** NADP-MDH, NADP-malate dehydrogenase; **(D)** PEPC, phosphoenolpyruvate carboxylase; **(E)** PPDK, pyruvate, phosphate dikinase.

It is currently estimated that C_4_ photosynthesis has arisen in at least 62 independent lineages of angiosperms (Sage et al., 2011). The polyphyletic origin of C_4_ photosynthesis indicates that only relatively small evolutionary changes were required for the establishment of this photosynthetic pathway (Gowik and Westhoff, 2011; Williams et al., 2013). The evolution of a novel C_4_ pathway is based on the creation of new genes, or functional changes in existing genes. Gene duplication has been recognized as one of the principal mechanisms of the evolution of new genes. Genes encoding enzymes of the C_4_ cycle often belong to gene families having multiple copies. For example, the *de novo* assembled four *MlPPDKs* in *M. lutarioriparius* was found, like the five *PPDKs* found in *M.* × *giganteus* (Naidu et al., 2003). When *M. lutarioriparius* transcripts of each gene family were put into the phylogenetic background of *Z. mays, S. bicolor, O. sativa*, and *A. thaliana* (Wang et al., 2009), one unique sequence of *M. lutarioriparius* in family CA, PEPC, NADP-MDH, NADP-ME, and PPDK was clustered with C_4_ orthologs in *Z. mays* and *S. bicolor* to form a remarkably long branch (Figure 3), suggesting that they are rapidly evolving compared to the other transcripts and those five orthologs would also encode C_4_ enzymes. All phylogeny analyses of C_4_ transcripts in five gene families were consistent with the single origin of NADP-ME type C_4_ photosynthesis in *Miscanthus, Sorghum*, and *Zea* (Giussani et al., 2001).

A total of 23 transcripts including 18 transcripts of Calvin cycle, and five transcripts of C_4_ cycle were identified to be involved in dark reaction.

### Population expression pattern of transcripts in light and dark reactions

The expression median FPKM of those nuclear encoded transcripts in dark reaction ranged from 14.3 to 12301.1 in JH and from 23.8 to 17476.6 in QG, and that of 30 transcripts in non-dark reaction from 15.2 to 3120.9 in JH and from 12.1 to 3885.6 in QG (Table S3). The expression distribution of transcripts in dark reaction significantly shifted to the high level relative to that in non-dark reaction both in JH and QG (Two-sample Kolmogorov–Smirnov test, *P* < 0.001; Two-sample Kolmogorov–Smirnov test, *P* < 0.0001). 91.3% of transcripts in dark reaction were up-regulated, while 53.3% of transcripts in non-dark reaction were down-regulated from JH to QG. The expression change of transcripts in dark reaction significantly shifted to the large fold relative to that in non-dark reaction (Two-sample Kolmogorov–Smirnov test, *P* = 0.0026, Figure S2). The median FPKM of degraded *MlrbcL*, the only chloroplast encoded transcript of dark reaction, slightly increased by 0.1 fold from JH to QG.

**Figure 4:**
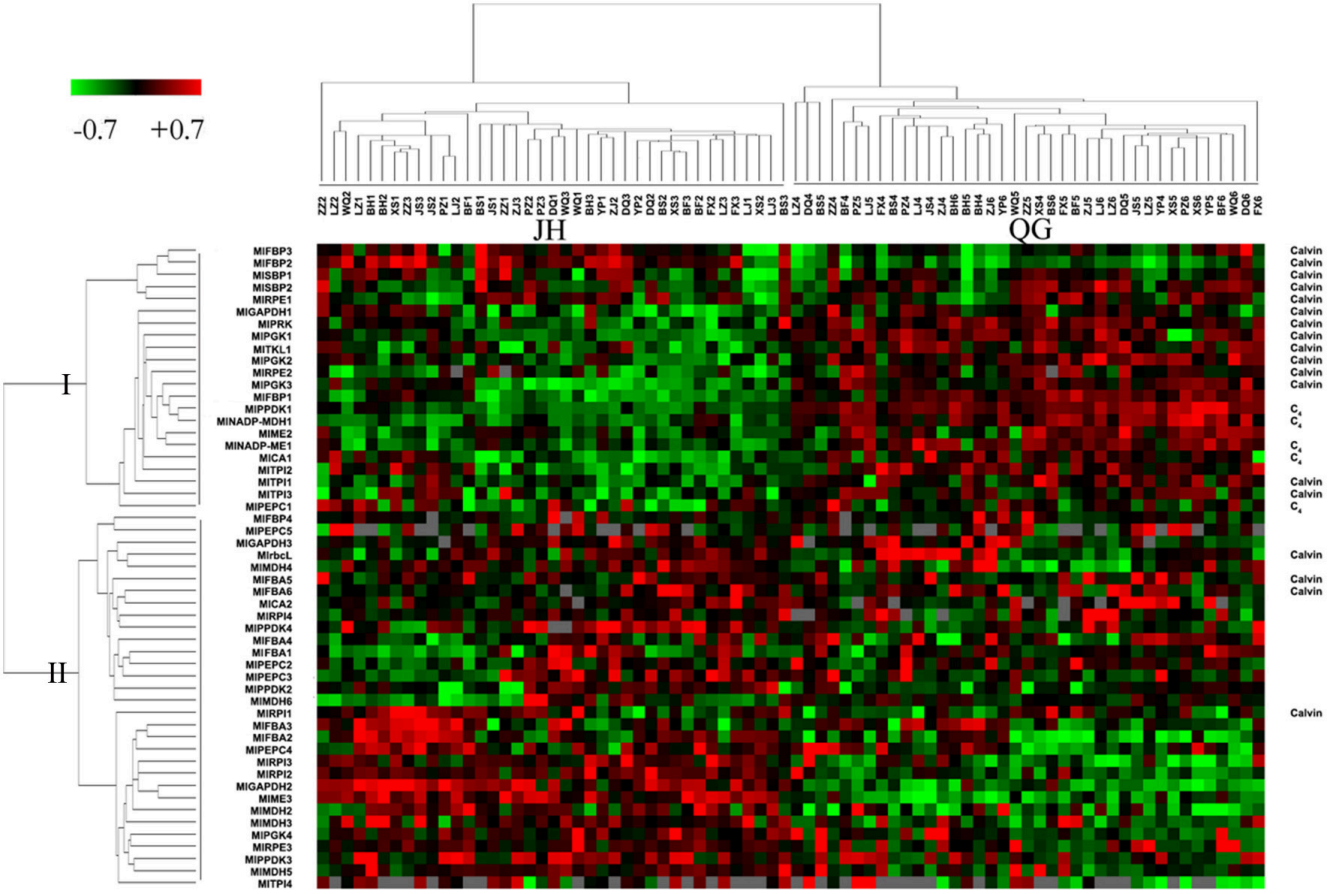
**Clustering of normalized gene expression profiles of dark reaction**. Each row represents data for one transcript, and each column represents data for one individual sampled from JH or QG. Expression levels for each transcript are depicted relative to the median level across all experimental samples and across all transcripts after log_2_ transformation (green, low levels; red, high levels; gray, no detected expression). Transcripts and samples are arranged by hierarchical clustering. The branch lengths of the cluster trees represent the degree of expression similarity from the highest to the lowest. The transcripts of C_4_ enzyme and Calvin enzyme were indicated in the right column.

Fifty-five genes in light reaction and fifty-three genes in dark and non-dark reactions were clustered using the expression profile of two sites, respectively. Both clustering maps at the sample dimension showed two distinct groups matching JH samples and QG samples, and at the gene dimension showed two and three big branches, respectively (Figures 4, S3). All 14 chloroplast encoded transcripts of light reaction were clustered into the Branch III, showing that their degradation was tightly coordinated and increased from JH to QG (Figure S3). The expression median FPKM ranged from 49.6 to 12301.1 in JH and from 103.0 to 17476.6 in QG within the Branch I and ranged from 14.3 to 3120.9 in JH and from 12.1 to 3885.6 in QG within the Branch II, which showed the expression level in the Branch I was larger than that in the Branch II and expression was up-regulated in the Branch I (Figure 4). *MlSBPs, MlPRKs*, and *MlTKLs* were only present in the Branch I; *MlrbcL, MlRPIs*, and *MlFBAs* were only present in the Branch II; multiple genes in other pathway positions were distributed in both Branch I and Branch II (Figure 4). 38 transcripts being harbored in ten gene families were present in Branch I and Branch II of the expression profile (Figure 4), which showed different expression patterns among these transcripts of the same gene family. Besides, two *MlFBPs* and two *MlSBPs* were clustered into one minor branch in Branch I, showing their possible functional similarity matches their sequence similarity (Figures 2G, 4). All five C_4_ transcripts and 77.8% of Calvin transcripts took up 86.4% of Branch I, which indicated the tight connection of each enzyme at the transcriptional level within the C_4_ cycle or the Calvin cycle and between the two cycles relative to their paralogs involved in other reactions, just like glycolysis and gluconeogenesis (Figure 4).

### The coordination of light and dark reactions by comparing population expression of two contrasting environments

**Figure 5:**
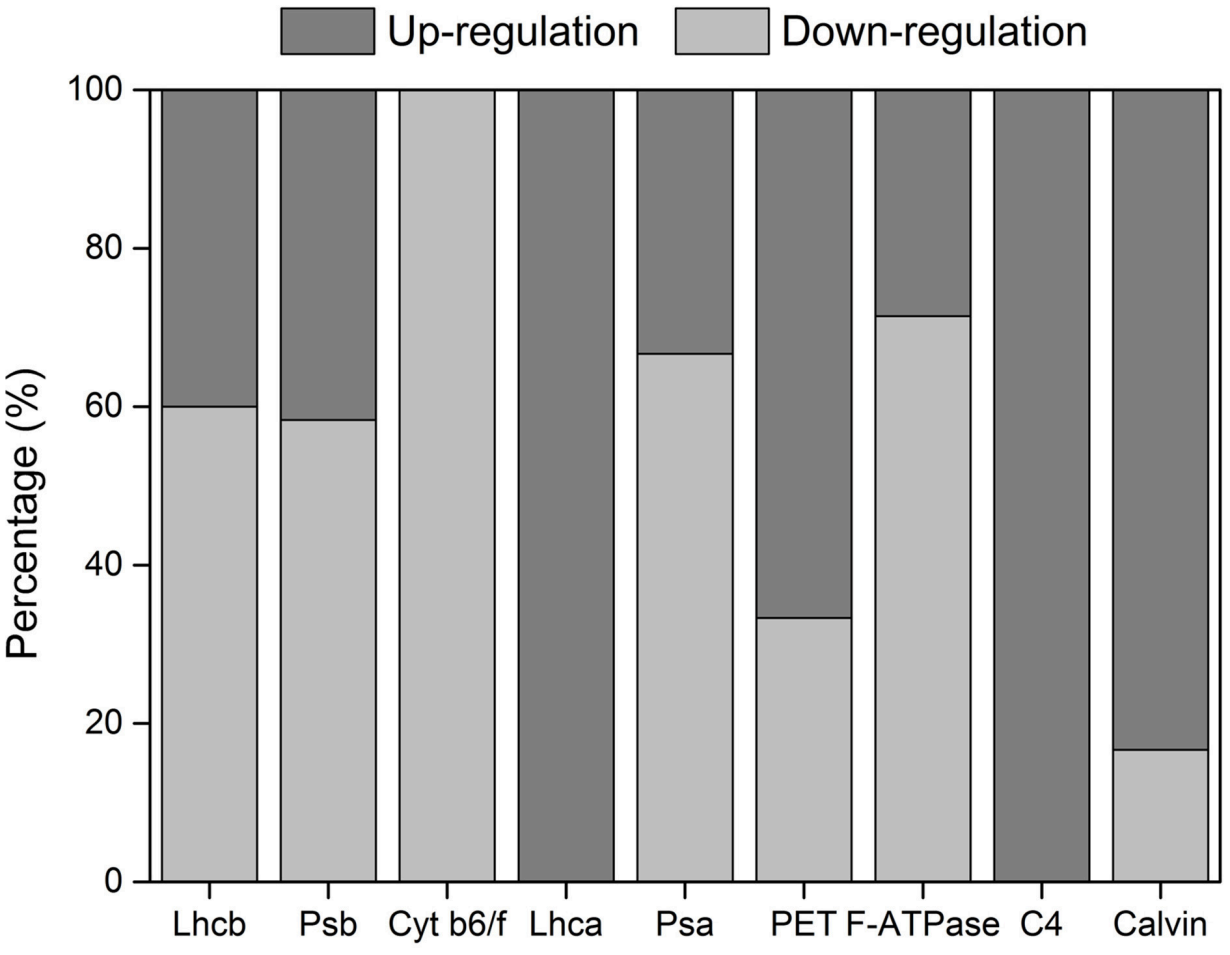
**The percentage of gene up-regulation and down-regulation in each functional group in QG in relative to JH**.

**Figure 6:**
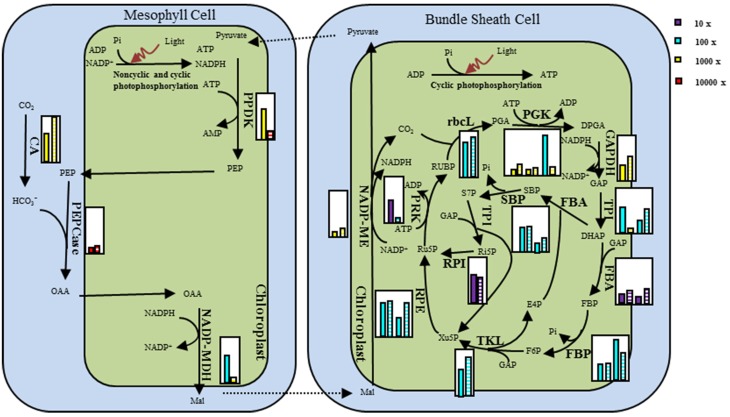
**Schematic view of difference in the NADP-ME type C_4_ pathway gene expression for *M. lutarioriparius* population in JH and in QG**. Relative transcript abundances are given in small inset white boxes. The transcript levels were represented by the median of the population in each site. The expression values were arranged in pairs with JH in the left and QG in the right. The expression value was indicated by the product of the color and the height of the histogram. The four colors represent four scale units, while the height of histogram was relative to the height of the inset box set as 10.

The transcripts in dark reaction and light reaction were used to analyze the coordination of each transcript within or between light and dark reaction pathways in facing the environment change. In light reaction, all seven function groups, defined according to the protein complexes in KEGG pathway, had more transcripts in being down-regulated than up-regulated, except for Lhca and PET groups, while all C_4_ transcripts and 83.3% of Calvin transcripts were up-regulated in dark reaction (Figure 5). Among those transcripts, 10 transcripts in light reaction were down-regulated by two-fold change and four transcripts including *MlPPDK2_PPDK1, MlPRK, MlPGK3*, and *MlFBA7* in dark reaction were up-regulated by two-fold change (Table S3). The great details of transcript expression change of the related pathways were shown in Figure 6. The NADP-ME type dark reaction pathway were made up of components expressed in mesophyll cells (MC) and bundle sheath cells (BSC), while the Calvin cycle pathway was expressed only in BSC. The expression level in NADP-ME type C_4_-specific pathway was significantly higher than those of Calvin cycle when *Miscanthus* population transplanted from the planting site near its native habit to Loess Plateau (Wilcoxon rank sum test, *P* < 0.0001; Figure 6).

**Figure 7:**
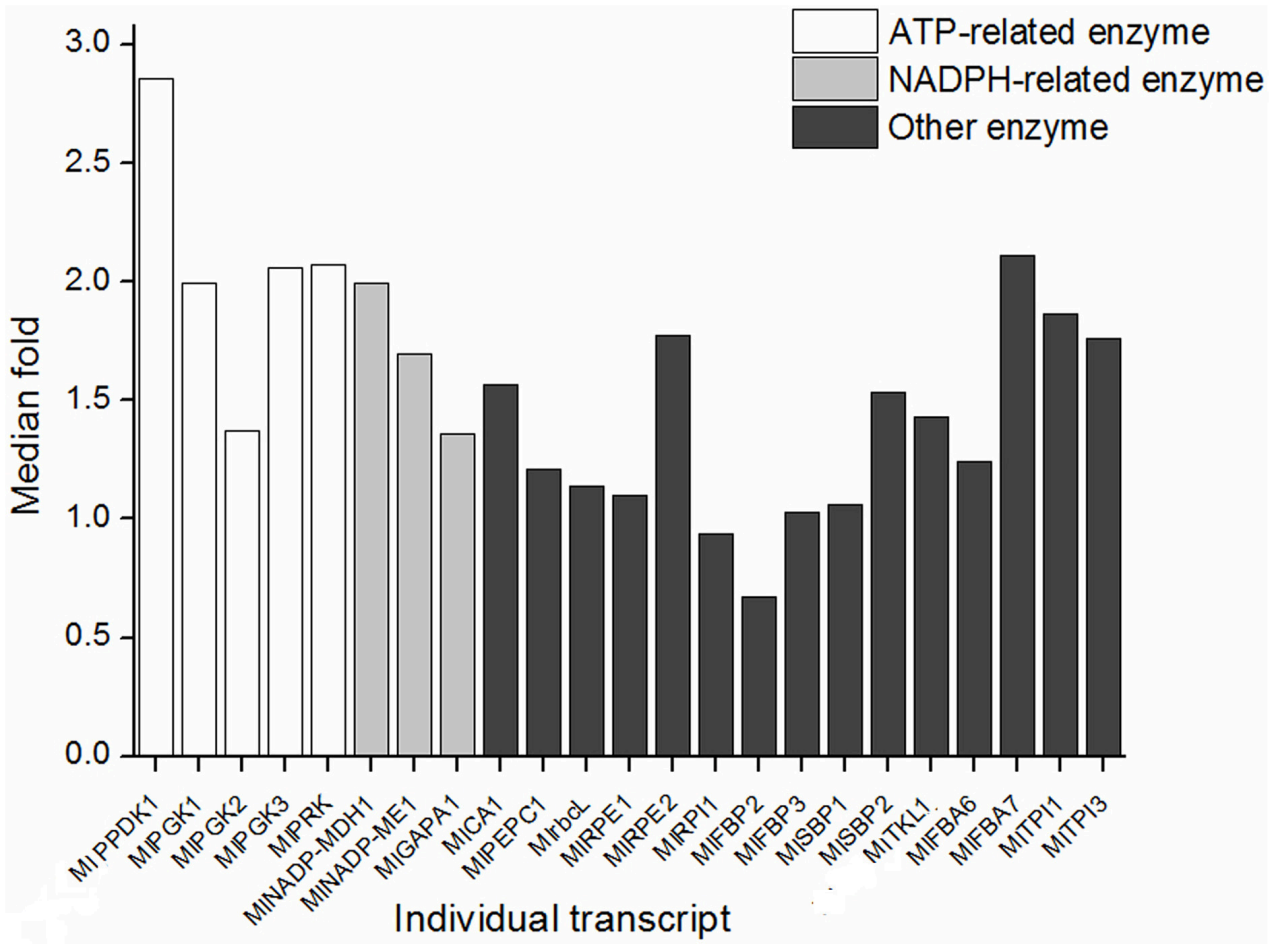
**Expression change of the ATP-related and NADPH-related transcripts between JH and QG**. The transcriptional levels were represented by the median of the population in each site. The height of histogram was calculated as the ratio of the transcriptional level in JH and in QG. ATP-related and NADPH-related mean the reaction needs input of the enzyme and input or output of ATP or NADPH.

Among those enzymes involved in dark reaction, PPDK, PGK, and PRK function with the consumption of ATP; NADP-MDH, GAPDH, and NADP-ME function with NADP+/NADPH cycle. Accordingly, transcripts associated with dark reaction were clarified as three groups, ATP-related, NADPH-related, and Others groups (Figure 7). The expression fold change of transcripts in ATP-related group was significantly larger than Others group (Wilcoxon rank sum test, *P* = 0.0232), while NADPH-related group was not significantly larger than Others group (Wilcoxon rank sum test, *P* = 0.3).

The distribution of expression correlation of each transcript pair in JH and QG was at the median of 0.16 (Figure S4). The average correlation of each transcript, indicating the potential interaction of other transcripts to this transcript as the center showed *MlatpH, MlNADP-ME1, MlatpA, MlatpF, MlpsaN, MlpsaI, MlPPDK1, MlPGK1*, and *MlPRK* were highly centered (Table S4). The expression similarity between and within each component of the photosynthesis pathway was showed in Table 1. In light reaction, any two of LHCI, PSI, and F-ATPase were significantly correlated with each other. In dark reaction, C_4_ cycle was significantly correlated with Calvin cycle. Between light and dark reactions, F-ATPase, and LHCI were both significantly correlated with C_4_ and Calvin cycles, meanwhile, PSII was significantly correlated with C_4_ cycle. Besides, the degradation of 14 chloroplast encoded transcripts including four PSII, 2 Cyt b6/f, 3 PSI, 5 F-ATPase were all tightly clustered (Figure S3; Table S3). In summary, 61 transcripts of six functional units, LHCI, PSI, PSII, F-ATPase, C_4_ cycle, and Calvin cycle, as well as two chloroplast encoded Cyt b6/f transcripts were coordinated under changing environments, which took up 80.8% of 78 photosynthetic transcripts.

**Table 1:** **Average correlation coefficient between and within each functional component of photosynthesis pathway**.

		**C_4_ (5)**	**Calvin (18)**	**Cyt *b_*6*_/f* (5)**	**F-ATPase (7)**	**PET (12)**	**Lhca (5)**	**Psa (9)**	**Lhcb (5)**	**Psb (12)**
**C**_4_**(5)**	0.0010	0.58^***^	0.40^***^	0.18	0.48^***^	0.27	0.33^*^	0.31	0.25	0.32^*^
**Calvin (18)**	0.0075	< 0.0001	0.31^**^	0.22	0.35^***^	0.22	0.31^**^	0.27	0.19	0.24
**Cyt** ***b**_*6*_**/f*** **(5)**	0.8280	0.9930	0.9870	0.26	0.29	0.18	0.19	0.25	0.17	0.27
**F-ATPase (7)**	0.0130	0.0003	< 0.0001	0.9550	0.54^*^	0.24	0.35^***^	0.37^**^	0.26	0.33
**PET (12)**	0.9990	0.5510	0.9920	0.9540	0.2230	0.17	0.22	0.20	0.14	0.20
**Lhca (5)**	0.0010	0.0220	0.0020	0.6550	0.0003	0.9230	0.46^***^	0.36^***^	0.22	0.20
**Psa (9)**	0.3090	0.1860	0.1540	0.8840	0.0030	0.9790	0.0004	0.31	0.23	0.26
**Lhcb (5)**	0.9890	0.5000	1.000	0.9980	0.2500	1.000	0.9930	0.9820	0.13	0.19
**Psb (12)**	0.9460	0.0260	0.5270	0.8170	0.0510	1.000	0.9740	0.6140	0.9930	0.24

The number of transcripts is shown in the bracket. The left-justified number is the average coefficient of each group, and the right-justified number with shading is the P-value (Binomial Test) between groups. The second column shows the P-value (Binomial Test) within each group.

* Indicates p < 0.05;

** indicates p < 0.01;

*** indicates p < 0.001.

## Discussion

Although, *M. lutarioriparius* is native to the seasonal flooding habitats along the Yangtze River, it surprisingly preserved high photosynthesis and WUE after being transplanted to the Loess Plateau where the annual precipitation is less than half of its native habitat (Yan et al., 2015b). The populations established differ between the two sites with *M. lutarioriparius* genetic diversity being less in the non-native site and expression diversity being greater in the non-native site (Xu et al., 2015). However, the genetic diversity distribution of photosynthetic genes in QG was not significantly different from that in JH (Table S5; Two-sample Kolmogorov–Smirnov test, *P* = 0.8288). Thus, we assumed that the selection pressure during establishment of seed populations at the non-native site could be ignored to account for the transcriptional differences on photosynthetic genes. To understand how C_4_ photosynthesis functioned in *M. lutarioriparius*' acclimating to changing environments, we explored the change and correlation of different elements within C_4_ photosynthesis by investigating population-wide expression of the light reaction and dark reaction across two contrasting environments. These analyses at the transcriptional level suggest that the coordination patterns within or between light and dark reactions provide raw materials for *M. lutarioriparius* in optimizing photosynthesis rate under the condition of reduced open degree of stoma. Compared to previous studies, how do these findings reveal the coordination of expression can increase photosynthetic plasticity of a C_4_ energy crop to changing environments?

### Different expression patterns of CET and LET across changing environments

In the NADP-ME type C_4_ plants, photosynthetic activities are portioned between two morphologically and biochemically distinct bundle-sheath and mesophyll chloroplasts (Majeran and van Wijk, 2009). Like maize and sorghum, the bundle-sheath chloroplasts of *M. lutarioriparius* have depleted PSII while mesophyll chloroplasts have complete PSII. The discordant expression change of PSII antenna proteins and PSII core proteins may reflect their different sensitivity of sensing environment change in MC, while the high correlation of up-regulated PSI antenna proteins and PSI core proteins indicated their relatively stable connection mainly in BSC (Pfundel and Neubohn, 1999; Majeran and van Wijk, 2009). LHCI and PSI groups both were not significantly correlated with LHCII and PSII groups, which indicated that expression regulation of PSI and PSII by the redox and energy state in photosynthetic cells was not the same (Pfannschmidt et al., 2009). Moreover, the low correlations between or within photosynthetic electron transport (PET) and cytochrome *b*_6_*/f* complex (cyt *b*_6_*/f*) may reflect the changed usage between CET around PSI and LET using both PSI and PSII in the new environment (Allen, 2003; Kramer et al., 2004; Lintala et al., 2007; Rumeau et al., 2007; Majeran and van Wijk, 2009).

According to ATP supply, the previous studies had revealed the importance of CET around PSI in C_4_ plants (Herbert et al., 1990; Asada et al., 1992). Consistent with the movement of metabolites between two cell types, the genes related to CET are up-regulated in BSC, just like the up-regulation of genes associated with LET in MC (Kubicki et al., 1994). In NADP-ME-type C_4_ plants, the NDH complex representing one route for CET accumulated more in BSC than in MC. Meanwhile, in NAD-ME-type C_4_ plants, more ATP is required in MC, and the NDH complex is over accumulated in MC. In contrast to the cell-type-specific accumulation of the NDH complex, PGR5, representing another route for CET is equally accumulated both in MC and BSC (Takabayashi et al., 2005). The CET could provide two additional ATPs for C_4_ plants to fix one CO_2_ through the concentrating mechanism, which minimizes the energy loss resulting from the oxygenase reaction of Rubisco. CET via NDH greatly expressed in the related cell with a strong need for ATP plays a central role in driving the CO_2_-concentrating mechanism in C_4_ photosynthesis (Takabayashi et al., 2005). Consistent with the increased degradation of 14 chloroplast encoded transcripts (Figure S3), all seven detected chloroplast encoded *Mlndhs* showed increased degradation above 1.26-fold, and at most the degradation of *MlndhH* was increased up to 2.55-fold in the Loess Plateau (Table S6), indicating that expression of transcripts related with CET was coordinated in changing environments. Furthermore, any two of the four groups consisting of CET, LHCI, PSI and F-ATPase in the light reaction (Figure 5), was significantly correlated with another one; while this was not the case in any two of the other three groups, LHCII, PSII, and F-ATPase (Table 1), which indicated that the expression of functional units are more tightly regulated in CET than in LET. The CET could also complement LET in balancing the ratio of ATP/NADPH required for CO_2_ fixation changes in different environments, depending on activities of photorespiration and nitrate assimilation to glutamate (Kramer et al., 2004). The highly flexible regulation of light reactions in chloroplasts can optimize energy production to downstream metabolism.

### Evolutionary insights into expression patterns in dark reaction

The distinct expression levels, fold change, and clusters of *M. lutarioriparius* enzymes in the Calvin cycle and non-Calvin cycle all support the identification of Calvin enzymes based on polyphyletic origin of the nuclear encoded and plastid targeted nature (Figures 2, 4; Figure S2).The isoforms of each gene family showed different expression patterns, and the five identified C_4_ transcripts were more highly expressed in leaf than their paralogs, and being clustered into Branch I (Figure 4). Some gene families involved in Calvin cycle allocated isoforms in both Branch I and Branch II (Figure 4). Compared to the expression level in JH, the expression values of C_4_-type *MlPPDK1* were 2.9-folds change in QG; while those of *MlrbcL* and *MlPEPC1* were 1.1- and 1.2-folds change (Figure 7). The similar expression change of those C_4_ photosynthetic enzymes were reported for not the gene expression level but the protein contents in the cold tolerance of *M.* × *giganteus* (Naidu et al., 2003; Wang et al., 2008b). Similarly, *M. lutarioriparius* elevated the expression of *MlPPDK1* for maintaining carbon assimilation under the cold, water-limited and high-light Loess Plateau.

The expression pattern of *M. lutarioriparius* genes in dark reaction showed the expression level in the C_4_ cycle was larger than in the Calvin cycle, although C_4_ cycle was significantly correlated with Calvin cycle (Figure 6; Table 1). The different responses between C_4_ cycle and Calvin cycle were reported for C_4_ plants facing water stress, where great leakiness of CO_2_ from the bundle sheath cell is induced (Ghannoum, 2009). One study showed that in the *Z. mays* leaf developmental gradient increase of transcripts representing subunits of the reductive pentose phosphate pathway was much less pronounced than that of the C_4_ transcripts (Pick et al., 2011). In the C_4_ cycle, *MlPPDK1* ranked top in gene expression change, followed by *MlNADP-ME1* and *MlPEPC1* at last (Figures 6, 7). Their response patterns were consistent with the results from the maize leaf developmental gradient (Li et al., 2010; Pick et al., 2011). Also, the increased fold of transcript abundance was larger for ATP-related group than NADPH-related group and Others group, which indicated the regulation of transcripts in C_4_ and Calvin cycles are both greatly sensitive to the energy state of photosynthetic cells (Figure 7).

### Acclimation potential from the coordination of light reaction and dark reaction at transcription level

For C_3_ plants, gene expression is coordinated between the chloroplast and the nucleus (Gray et al., 2003; Woodson and Chory, 2008; Berry et al., 2013). On one hand, the retrograde signals from the chloroplast are master switches of transcriptional regulation of 71 nucleus-encoded chloroplast-localized proteins of *A. thaliana* under 23 different genetic and environmental conditions (Richly et al., 2003). On the other hand, the anterograde signals from the nucleus lead to eight sets of co-regulated chloroplast genes across 89 *A. thaliana* chloroplast transcriptomes (Cho et al., 2009). For C_4_ plants, the regulation of gene expression is also coordinated for adjusting the photosynthetic capacity in photosynthetic cells when growing under a particular set of conditions (Woodson and Chory, 2008; Pfannschmidt et al., 2009). For example, the expression change of lhcb5, ndhF, atpA, psbA, petA, and lhcb4 in light reaction was detected in *M.* × *giganteus* after 14 days of chilling (Spence et al., 2014). The amount of PPDK, rbcL, ATPase beta subunit was up-regulated in the leaf proteome as heat-response proteins of *M. sinensis* (Sharmin et al., 2013). In contrast with the acclimation experiments to single controlled environment factor (Sun et al., 2012, 2014; Sharmin et al., 2013), 80.8% of *M. lutarioriparius* photosynthetic transcripts were coordinated at transcription level across two contrasting field environments.

Although, a large gap exists in understanding the relationship between the coordinated gene expression and coordinated metabolic process, it is certain that coordination at transcription level was an important mechanism of adjusting photosynthetic capacity (Woodson and Chory, 2008). The coordinated degradation of chloroplast encoded transcripts in light reaction and expression of nuclear encoded transcripts in dark reaction indicates the light-driven redox chemistry of the light reactions and the temperature-dependent enzymatic reactions of the dark reaction are tightly coupled (Pfannschmidt et al., 2009). The coordinated expression of LHCI, PSI, and F-ATPase indicates that the potential determinant is related to the cyclic photophosphorylation. The cyclic photophosphorylation can regulate the ratio of ATP and NADPH (Allen, 2003). The sufficient ATP supply strengthened the CO_2_ pump for carbon assimilation by using NADP-ME type C_4_-specific pathway only consuming net ATP and no NADPH (Wang et al., 2011, 2014). *M. lutarioriparius* appeared to coordinate most transcripts of light and dark reactions across changing environments by adjusting carbon assimilation, which seemed to be powered by cyclic electron transport.

Analysis of the degree of coordinated expression within photosynthesis pathway revealed valuable insights into the potential plasticity of plant acclimation to changing environments. In combination with the degree of coordinated metabolic process, this will contribute to further understanding of complex photosynthetic plasticity of *Miscanthus* in changing environments.

## Author contributions

TS, JY, and JL conceived and designed the experiments. SX, LK, JY, QX, YF, ZS, QW, and CZ performed the experiments. SX, LK, QX, CZ, WL, YF, and TS performed data analysis. SX, LK, QX, JY, and TS wrote the manuscript. All authors read and approved the final manuscript.

## Funding

The work was supported by grants from the Key Program of the National Natural Science Foundation of China (NSFC91131902), the National Science and Technology Project for Rural Development of the Twelfth Five-year-Plan of China (2013BAD22B02), and Science and Technology Service Network Initiative (KFJ-EW-STS-119, KFJ-EW-STS-061).

### Conflict of interest statement

The authors declare that the research was conducted in the absence of any commercial or financial relationships that could be construed as a potential conflict of interest. The reviewer Xinguang Zhu and Handling Editor declared their shared affiliation, and the Handling Editor states that the process nevertheless met the standards of a fair and objective review.

## Abbreviations

BSC: bundle sheath cells
CA: carbonic anhydrase
CET: cyclic electron transport
cyt *b*_6_*/f*: cytochrome *b*_6_*/f* complex
FBA: fructose- bisphosphate aldolase
FBP: fructose bisphosphatase
GAPDH: glyceraldehyde-3-phosphate dehydrogenase
JH: Jiangxia of Hubei Province
LET: linear electron transport
LHCI: light-harvesting chlorophyll-a/b protein of photosystem I
MC: mesophyll cells
NADP-MDH: NADP-malate dehydrogenase
NADP-ME: NADP-malic enzyme
PET: photosynthetic electron transport
PEPC: phosphoenolpyruvate carboxylase
PGK: Phosphoglycerate Kinase
PPDK: pyruvate phosphate dikinase
PRK: Phosphoribulokinase
PSI: photosystem I
QG: Qingyang of Gansu Province
rbcL: Rubisco large subunit
RPE: D-ribulose-5-phosphate 3-epimerase
RPI: ribose-5-phosphate isomerase
SBP: sedoheptulose-bisphosphatase
TKL: transketolase
TPI: triose phosphate isomerase
WUE: water use efficiency.
