# Supplementary figures and images for: The Coordination of Gene Expression within Photosynthesis Pathway for Acclimation of C4 Energy Crop Miscanthus lutarioriparius
Source: Front Plant Sci. 2016 Feb 9;7:109. doi: 10.3389/fpls.2016.00109 (PMC4746358; doi:10.3389/fpls.2016.00109)

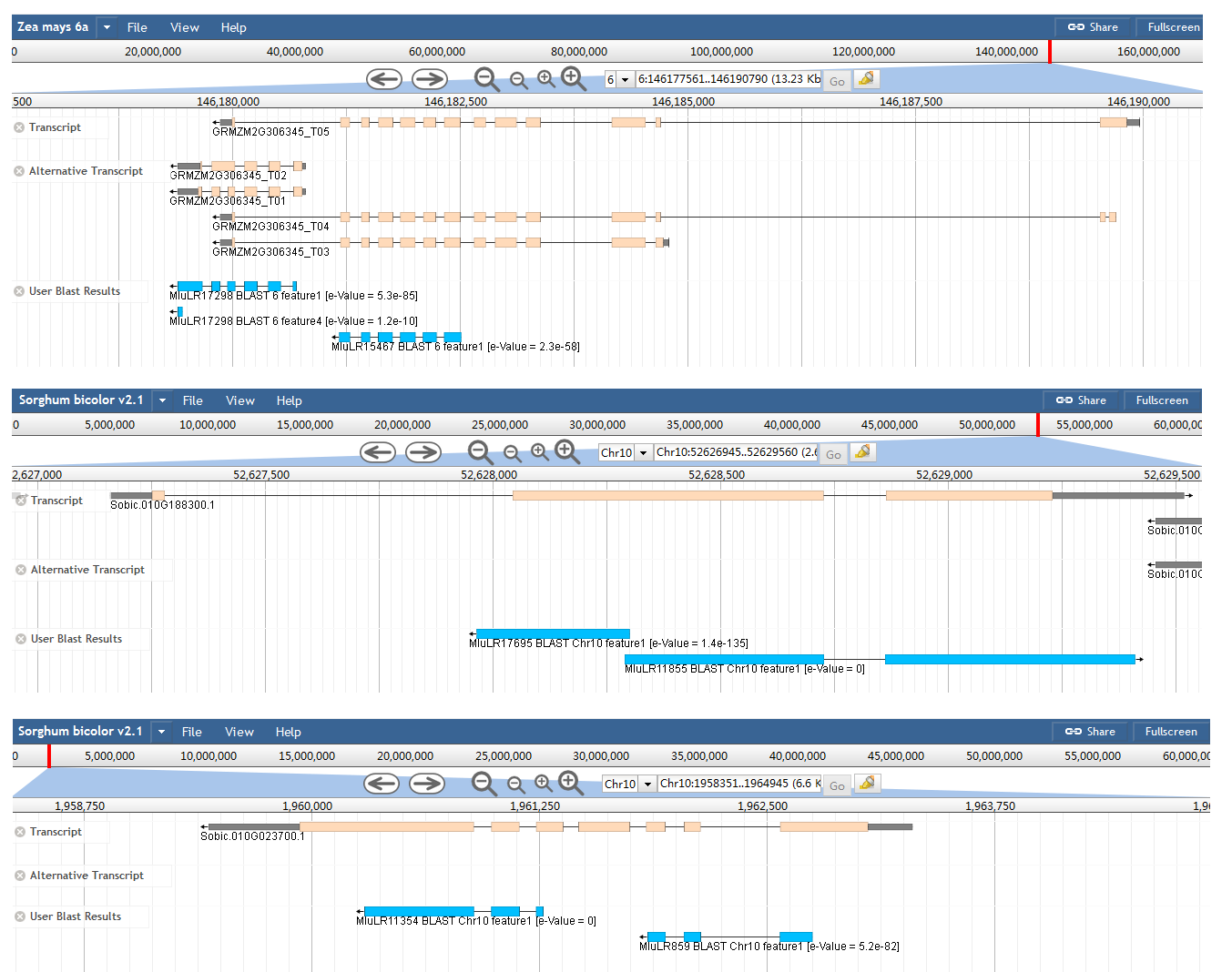

Supplement: Supplementary file 2 [file Image1.TIF]

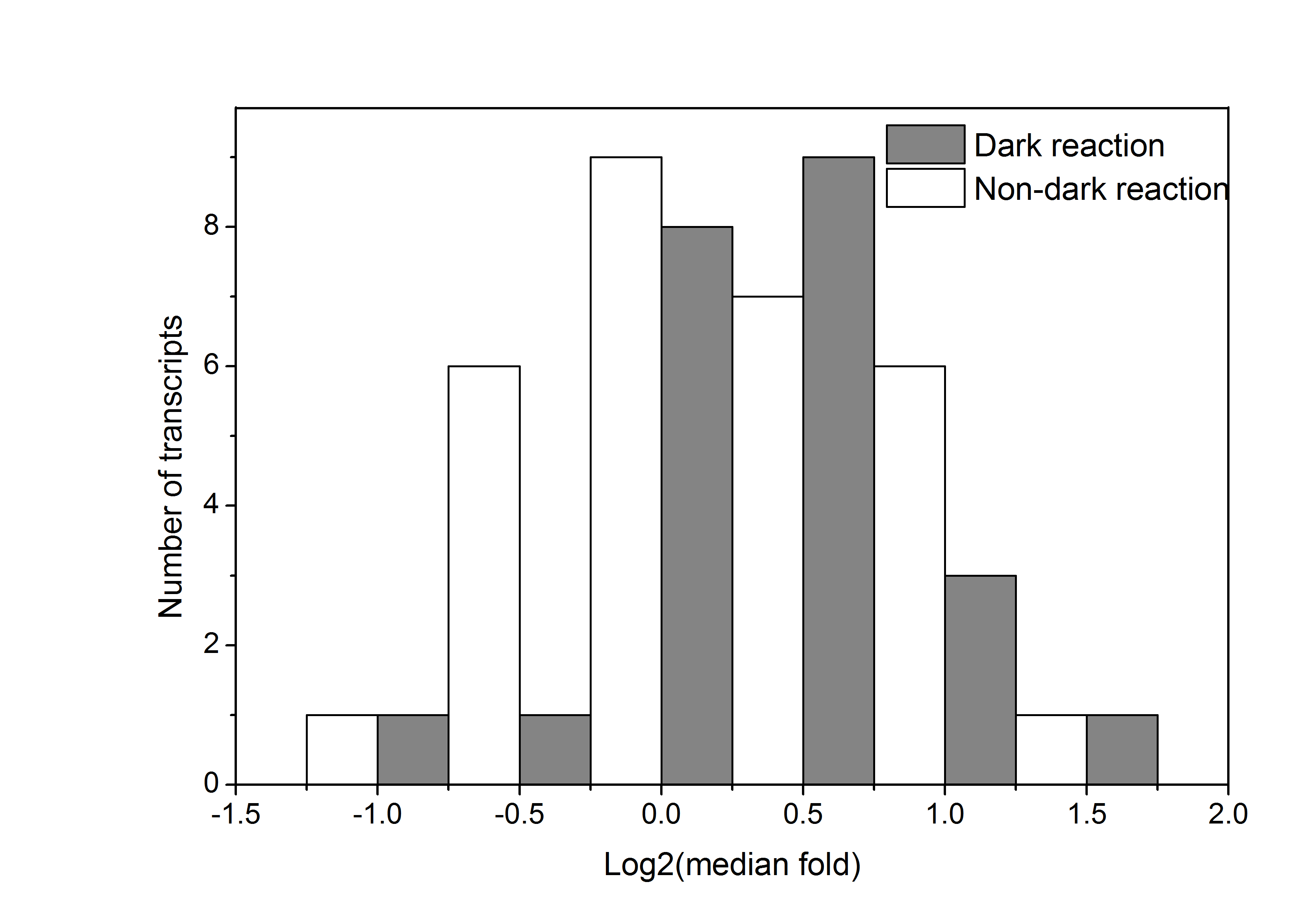

Supplement: Supplementary file 3 [file Image2.PNG]

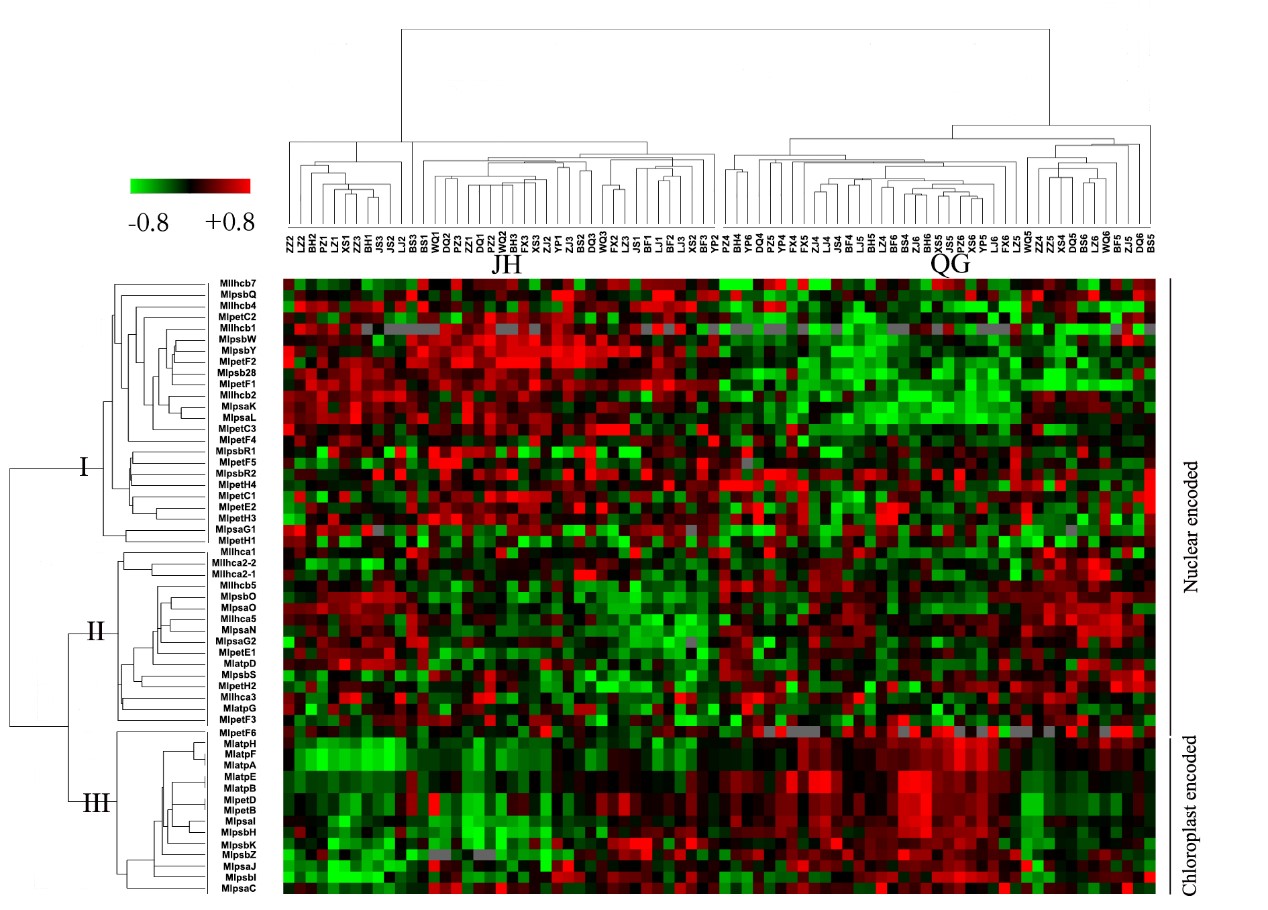

Supplement: Supplementary file 4 [file Image3.JPEG]

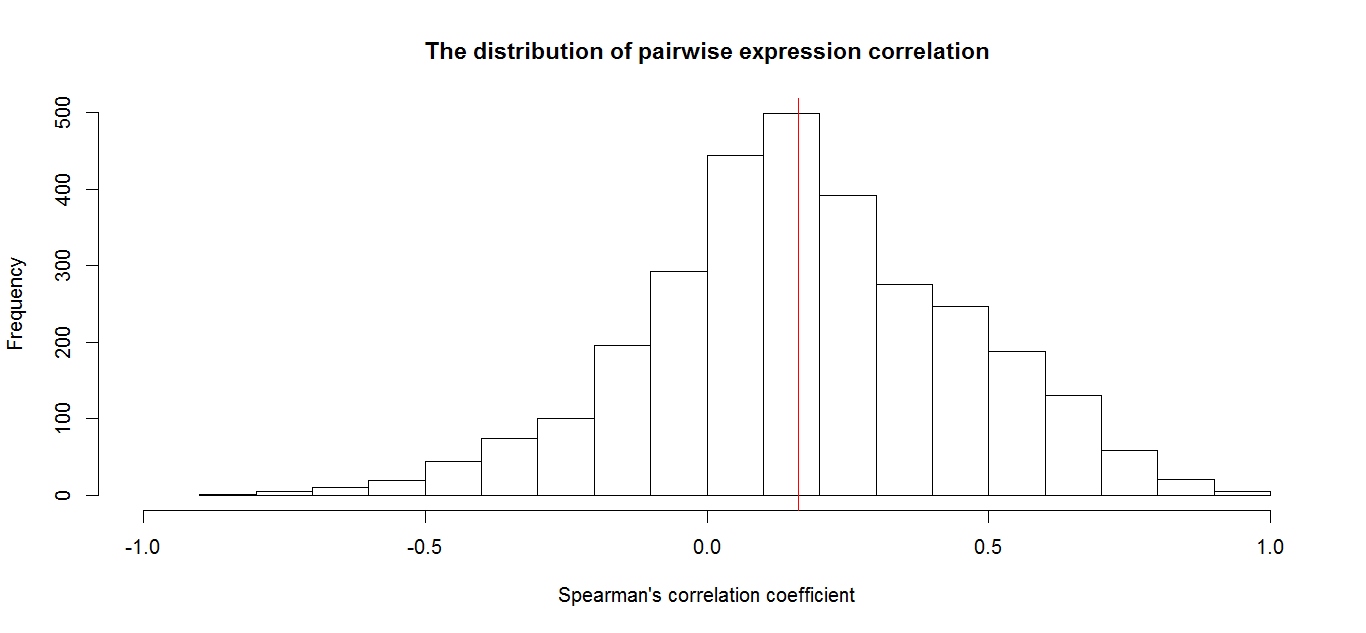

Supplement: Supplementary file 5 [file Image4.JPEG]
